# Bis[1,3-bis­(2,4,6-trimethyl­phen­yl)-2,3-dihydro-1*H*-imidazol-2-yl­idene]dichloridodinitro­syltungsten(II) tetra­hydro­furan-*d*_8_ monosolvate

**DOI:** 10.1107/S1600536810050099

**Published:** 2010-12-04

**Authors:** Javier Fraga-Hernández, Olivier Blacque, Heinz Berke

**Affiliations:** a Institut of Inorganic Chemistry, University of Zürich, Winterthurerstrasse 190, 8057 Zürich, Switzerland

## Abstract

The mol­ecular structure of the title compound, [WCl_2_(NO)_2_(C_21_H_24_N_2_)_2_]·C_4_D_8_O, displays a distorted octa­hedral arrangement around the W atom with two *trans* 1,3-bis­(2,4,6-trimethyl­phen­yl)imidazol-2-yl­idene (IMes) carbene ligands in axial positions. The four equatorial positions are occupied by nitrosyl and chloride ligands, which are *trans* to each other. The C_carbene_—W—C_carbene_ bond angle of 173.44 (18)° and the Cl—W—N_nitros­yl_ bond angles of 171.34 (11) and 171.32 (13)° deviate only slightly from linearity. The distortion comes from the nitrosyl and chloride ligands which are not fully coplanar since the two N atoms deviate from the WCl_2_ plane by −0.279 (4) and 0.272 (4) Å, respectively. An inter­molecular C—H⋯O inter­action connects the organometallic mol­ecule and the tetra­hydro­furan-*d*_8_ solvent mol­ecule.

## Related literature

For the synthesis, characterization and reactivity of dinitrosyl tungsten complexes in various oxidation states, see: Fraga-Hernández (2007 ▶). For tungsten complexes with *N*-heterocyclic (NHC) carbenes, see: Nonnenmacher *et al.* (2005 ▶); Hahn *et al.* (2005 ▶); Wu *et al.* (2007 ▶). For an overview of the first organometallic nitro­syls, see: Enemark & Feltham (1974 ▶); Richter-Addo & Legzdins (1988 ▶); Berke & Burger (1994 ▶).
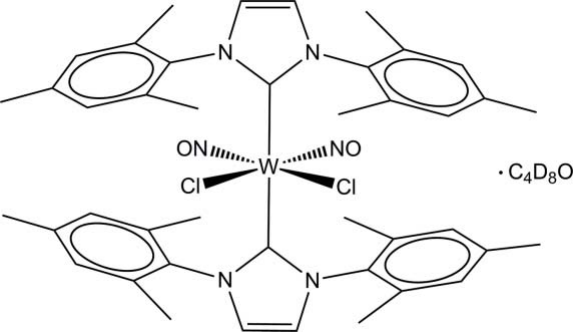


## Experimental

### 

#### Crystal data

[WCl_2_(NO)_2_(C_21_H_24_N_2_)_2_]·C_4_D_8_O
*M* *_r_* = 1003.72
Triclinic, 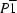

*a* = 11.3861 (7) Å
*b* = 13.0517 (9) Å
*c* = 17.0448 (11) Å
α = 81.245 (8)°
β = 72.473 (7)°
γ = 68.983 (7)°
*V* = 2251.9 (3) Å^3^
*Z* = 2
Mo *K*α radiation
μ = 2.73 mm^−1^
*T* = 183 K
0.25 × 0.15 × 0.08 mm

#### Data collection

Stoe IPDS diffractometer
Absorption correction: numerical (Coppens *et al.*, 1965 ▶) *T*_min_ = 0.568, *T*_max_ = 0.816
44725 measured reflections
7466 independent reflections
6365 reflections with *I* > 2σ(*I*)
*R*_int_ = 0.046

#### Refinement

*R*[*F* ^2^ > 2σ(*F* ^2^)] = 0.026
*wR*(*F* ^2^) = 0.076
*S* = 1.12
7466 reflections
535 parameters
H-atom parameters constrained
Δρ_max_ = 1.00 e Å^−3^
Δρ_min_ = −0.42 e Å^−3^

### 

Data collection: *EXPOSE* in *IPDS Software* (Stoe & Cie, 1999 ▶); cell refinement: *CELL* in *IPDS Software*; data reduction: *INTEGRATE* in *IPDS Software*; program(s) used to solve structure: *SHELXS97* (Sheldrick, 2008 ▶); program(s) used to refine structure: *SHELXL97* (Sheldrick, 2008 ▶); molecular graphics: *ORTEP-3 for Windows* (Farrugia, 1997 ▶); software used to prepare material for publication: *SHELXL97*, *WinGX* (Farrugia, 1999 ▶) and *publCIF* (Westrip, 2010 ▶).

## Supplementary Material

Crystal structure: contains datablocks global, I. DOI: 10.1107/S1600536810050099/fj2368sup1.cif

Structure factors: contains datablocks I. DOI: 10.1107/S1600536810050099/fj2368Isup2.hkl

Additional supplementary materials:  crystallographic information; 3D view; checkCIF report

## Figures and Tables

**Table 1:** Hydrogen-bond geometry (Å, °)

*D*—H⋯*A*	*D*—H	H⋯*A*	*D*⋯*A*	*D*—H⋯*A*
C2—H2⋯O3	0.93	2.40	3.320 (7)	172

**Fig. 1.**
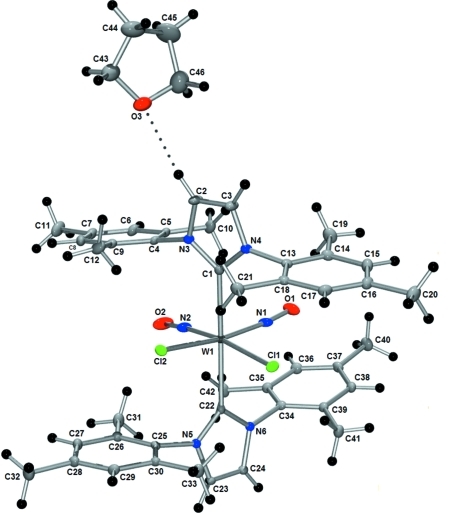
View of the title compound showing the labeling of the non-H atoms and 20% probability ellipsoids.

## supplementary crystallographic information

### Comment

In the course of our efforts on the synthesis of novel dinitrosyl hydride and dihydride tungsten derivatives bearing sterically demanding and highly donating phosphine ligands or N-heterocyclic (NHC) carbene ligands, the title compound W(NO)_2_Cl_2_(IMes)_2_.C_4_D_8_O was synthesized as an intermediate species by the reaction of the coordination polymer dinitrosyldichlorotungsten [W(NO)_2_Cl_2_]_n_ with 1,3-bis(2,4,6-trimethylphenyl)imidazol-2-ylidene (IMes), purified and characterized by several spectroscopic techniques and single-crystal X-ray diffraction.

The molecular structure of the title compound, C_42_H_48_Cl_2_N_6_O_2_W.C_4_D_8_O, displays a distorted octahedral arrangement around the W center with two *trans* 1,3-bis(2,4,6-trimethylphenyl)imidazol-2-ylidene ligands in axial positions, and the four equatorial positions are occupied by *trans* nitrosyl and chloride ligands (Fig. 1). The C_carbene_—W—C_carbene_ bond angle of 173.44 (18)° and the Cl—W—N_nitrosyl_ bond angles of 171.34 (11) and 171.32 (13)° deviates only slightly from linearity. The distortion comes from the nitrosyl and chloride ligands which are not fully coplanar since N1 and N2 deviate from the Cl1—W1—Cl2 plane by -0.279 (4) and +0.272 (4) Å, respectively. The five-membered rings of the carbene ligands are almost perpendicular to each other, the dihedral angle between the mean planes C1/N3/C2/C3/N4 and C22/N5/C23/C25/N6 is 74.2 (2)°. Each ring adopts an eclipsed conformation with one linear Cl—W—N_NO_ moiety, the dihedral angles between the mean planes Cl1/W1/N2/C1 and C1/N3/C2/C3/N4, and between Cl2/W1/N1/C22 and C22/N5/C23/C25/N6, are 15.8 (2) and 5.2 (2)°, respectively.

In the crystal structure, intermolecular C—H···O interactions connect the metal-organic molecules and the tetrahydrofurane-d_8_ solvent molecules (Table 1).

### Experimental

To a stirred suspension of [W(NO)_2_Cl_2_]_n_ (128.4 mg, 0.408 mmol) in 15 ml THF was added dropwise a solution of IMes = 1,3-bis(2,4,6-trimethylphenyl)imidazol-2-ylidene (248 mg, 0.815 mmol) in THF (5 ml) over a period of 15 minutes. Gradually, the solution intensified in color and a floculent black residue formed. The reaction mixture was stirred at room temperature for 6 h to obtain a deep green solution whose IR spectrum exhibited vibrations at 1737 and 1624 cm^-1^ attributable to nitrosyl groups. The volatiles were removed under vacuum, and the residue was extracted with 25 ml of toluene. The dark green solution was filtered through celite and dried under vacuum. The solid was recrystallized in CH_2_Cl_2_/pentane, leaving a fraction of green crystals, which where washed with pentane (2 *x* 3 ml) and dried *in vacuo* to afford 312.8 mg of [W(NO)_2_Cl_2_(IMes)_2_] (83%).

IR (ATR, 22°C, cm^-1^): 1737 (NO), 1624 (NO).

^1^H NMR (THF-d_8_, 300 MHz, 22°C): δ 6.97 (s, *NCH*, 4H, ^3^J_HH_ = 0.6 Hz), 6.78 (s, *ArH*, 8H), 2.25 (s, *4-CH_3_*, 12H), 1.99 (s, *2,6-CH_3_*, 24H).

^13^C {^1^H} NMR (benzene-d_6_, 125.8 MHz, 22°C): δ 182.6 (s, *NCN*), 139.6 (s, *Mes C-1*), 134.6 (s, *Mes C-4*), 129.8 (s, *Mes C-2,6*), 128.9 (s, *Mes C-3,5*), 122.4 (s, *NCC*), 21.0 (s, *4-CH_3_*), 17.4 (s, *2,6-CH_3_*).

Elemental analysis (%) calculated for C_42_H_48_Cl_2_N_6_O_2_W: C (54.62), H (5.24), N (9.10); found C (54.83), H (5.44), N (9.02).

### Refinement

All H positions were calculated after each cycle of refinement using a riding model with C—H = 0.93 Å for aromatic H atoms, with C—H = 0.96 Å for methyl H atoms, and with C—H = 0.97 Å for methylene H atoms [*U*_iso_(H) = 1.3*U*_eq_(C)].

### Crystal data

**Table:**

[WCl_2_(NO)_2_(C_21_H_24_N_2_)_2_]·C_4_D_8_O	*Z* = 2
*M**_r_* = 1003.72	*F*(000) = 1012
Triclinic, *P*1	*D*_x_ = 1.48 Mg m^−^^3^
Hall symbol: -P 1	Mo *K*α radiation, λ = 0.71073 Å
*a* = 11.3861 (7) Å	Cell parameters from 7997 reflections
*b* = 13.0517 (9) Å	θ = 3.0–30.3°
*c* = 17.0448 (11) Å	µ = 2.73 mm^−^^1^
α = 81.245 (8)°	*T* = 183 K
β = 72.473 (7)°	Plate, green
γ = 68.983 (7)°	0.25 × 0.15 × 0.08 mm
*V* = 2251.9 (3) Å^3^	

### Data collection

**Table:**

Stoe IPDS diffractometer	7466 independent reflections
Radiation source: fine-focus sealed tube	6365 reflections with *I* > 2σ(*I*)
graphite	*R*_int_ = 0.046
φ oscillation scan	θ_max_ = 25°, θ_min_ = 3.0°
Absorption correction: numerical (Coppens *et al.*, 1965)	*h* = −12→13
*T*_min_ = 0.568, *T*_max_ = 0.816	*k* = −15→15
44725 measured reflections	*l* = 0→20

### Refinement

**Table:**

Refinement on *F*^2^	0 restraints
Least-squares matrix: full	H-atom parameters constrained
*R*[*F*^2^ > 2σ(*F*^2^)] = 0.026	*w* = 1/[σ^2^(*F*_o_^2^) + (0.0434*P*)^2^] where *P* = (*F*_o_^2^ + 2*F*_c_^2^)/3
*wR*(*F*^2^) = 0.076	(Δ/σ)_max_ = 0.001
*S* = 1.12	Δρ_max_ = 1.00 e Å^−^^3^
7466 reflections	Δρ_min_ = −0.42 e Å^−^^3^
535 parameters	

### Special details

**Table:**

Geometry. All e.s.d.'s (except the e.s.d. in the dihedral angle between two l.s. planes) are estimated using the full covariance matrix. The cell e.s.d.'s are taken into account individually in the estimation of e.s.d.'s in distances, angles and torsion angles; correlations between e.s.d.'s in cell parameters are only used when they are defined by crystal symmetry. An approximate (isotropic) treatment of cell e.s.d.'s is used for estimating e.s.d.'s involving l.s. planes.

### Fractional atomic coordinates and isotropic or equivalent isotropic displacement parameters (Å^2^)

**Table:**

	*x*	*y*	*z*	*U*_iso_*/*U*_eq_	
W1	0.748163 (17)	0.774765 (17)	0.734179 (12)	0.02191 (7)	
Cl1	0.74039 (11)	0.82743 (12)	0.59143 (8)	0.0377 (3)	
Cl2	0.63266 (11)	0.96649 (11)	0.76621 (9)	0.0401 (3)	
N1	0.8115 (4)	0.6268 (4)	0.7123 (3)	0.0350 (10)	
O1	0.8406 (4)	0.5351 (3)	0.7034 (3)	0.0607 (13)	
N2	0.7786 (4)	0.7372 (4)	0.8350 (3)	0.0434 (12)	
O2	0.8123 (5)	0.7087 (5)	0.8966 (3)	0.0757 (17)	
C1	0.5416 (4)	0.7669 (4)	0.7755 (3)	0.0235 (10)	
N3	0.4669 (3)	0.7666 (4)	0.8548 (2)	0.0287 (9)	
C2	0.3430 (5)	0.7670 (5)	0.8590 (3)	0.0442 (15)	
H2	0.2756	0.7681	0.9065	0.057*	
C3	0.3397 (5)	0.7653 (5)	0.7825 (3)	0.0434 (15)	
H3	0.2693	0.7645	0.766	0.056*	
N4	0.4603 (3)	0.7649 (4)	0.7312 (2)	0.0286 (9)	
C4	0.5013 (4)	0.7590 (4)	0.9315 (3)	0.0258 (10)	
C5	0.5675 (4)	0.6562 (4)	0.9616 (3)	0.0302 (11)	
C6	0.5887 (5)	0.6493 (5)	1.0389 (3)	0.0381 (12)	
H6	0.6348	0.5816	1.0595	0.049*	
C7	0.5432 (5)	0.7401 (5)	1.0858 (3)	0.0399 (14)	
C8	0.4749 (5)	0.8402 (5)	1.0539 (3)	0.0393 (14)	
H8	0.4437	0.9018	1.085	0.051*	
C9	0.4517 (4)	0.8518 (4)	0.9777 (3)	0.0310 (12)	
C10	0.6143 (6)	0.5546 (5)	0.9139 (4)	0.0431 (13)	
H10A	0.5501	0.5573	0.8871	0.056*	
H10B	0.6279	0.4907	0.9508	0.056*	
H10C	0.695	0.5509	0.8732	0.056*	
C11	0.5614 (6)	0.7296 (7)	1.1711 (4)	0.062 (2)	
H11A	0.5813	0.7919	1.1796	0.081*	
H11B	0.6319	0.6635	1.1766	0.081*	
H11C	0.4826	0.7268	1.2112	0.081*	
C12	0.3677 (5)	0.9606 (5)	0.9488 (4)	0.0460 (15)	
H12A	0.2829	0.9572	0.9538	0.06*	
H12B	0.4075	0.9766	0.8923	0.06*	
H12C	0.3591	1.0174	0.982	0.06*	
C13	0.4801 (4)	0.7632 (4)	0.6427 (3)	0.0271 (11)	
C14	0.5257 (4)	0.6605 (4)	0.6083 (3)	0.0325 (12)	
C15	0.5311 (5)	0.6613 (5)	0.5253 (3)	0.0393 (14)	
H15	0.5628	0.5948	0.5	0.051*	
C16	0.4906 (4)	0.7580 (5)	0.4795 (3)	0.0371 (13)	
C17	0.4438 (4)	0.8572 (5)	0.5170 (3)	0.0352 (12)	
H17	0.4162	0.9224	0.4865	0.046*	
C18	0.4374 (4)	0.8611 (4)	0.5994 (3)	0.0279 (11)	
C19	0.5644 (6)	0.5538 (5)	0.6569 (4)	0.0484 (15)	
H19A	0.6345	0.5503	0.6786	0.063*	
H19B	0.5927	0.4938	0.6218	0.063*	
H19C	0.491	0.5489	0.7015	0.063*	
C20	0.4971 (5)	0.7543 (7)	0.3890 (4)	0.0550 (18)	
H20A	0.5867	0.7257	0.3579	0.071*	
H20B	0.4579	0.8271	0.3678	0.071*	
H20C	0.4509	0.7077	0.3844	0.071*	
C21	0.3868 (5)	0.9700 (4)	0.6394 (4)	0.0393 (13)	
H21A	0.3122	0.9721	0.6852	0.051*	
H21B	0.362	1.0287	0.6	0.051*	
H21C	0.454	0.9783	0.6584	0.051*	
C22	0.9457 (4)	0.8013 (4)	0.6851 (3)	0.0218 (10)	
N5	0.9786 (3)	0.8939 (3)	0.6796 (2)	0.0223 (8)	
C23	1.1098 (4)	0.8746 (4)	0.6391 (3)	0.0253 (10)	
H23	1.1533	0.9255	0.6276	0.033*	
C24	1.1615 (4)	0.7697 (4)	0.6197 (3)	0.0268 (11)	
H24	1.2479	0.7333	0.5926	0.035*	
N6	1.0612 (3)	0.7249 (3)	0.6478 (2)	0.0223 (8)	
C25	0.9052 (4)	0.9987 (4)	0.7170 (3)	0.0238 (10)	
C26	0.9017 (4)	1.0074 (4)	0.7986 (3)	0.0271 (11)	
C27	0.8452 (5)	1.1120 (4)	0.8294 (3)	0.0322 (12)	
H27	0.8398	1.1197	0.8839	0.042*	
C28	0.7966 (5)	1.2048 (4)	0.7820 (3)	0.0323 (11)	
C29	0.8005 (4)	1.1917 (4)	0.7015 (3)	0.0304 (11)	
H29	0.7662	1.2532	0.6695	0.04*	
C30	0.8543 (4)	1.0889 (4)	0.6676 (3)	0.0255 (10)	
C31	0.9567 (6)	0.9102 (5)	0.8509 (4)	0.0428 (14)	
H31A	0.8997	0.867	0.8675	0.056*	
H31B	0.9645	0.9345	0.8989	0.056*	
H31C	1.0412	0.8663	0.8201	0.056*	
C32	0.7440 (6)	1.3177 (5)	0.8157 (4)	0.0492 (15)	
H32A	0.713	1.3119	0.8747	0.064*	
H32B	0.6735	1.3634	0.7932	0.064*	
H32C	0.8122	1.3496	0.8008	0.064*	
C33	0.8575 (5)	1.0771 (4)	0.5809 (3)	0.0359 (12)	
H33A	0.9425	1.0721	0.5453	0.047*	
H33B	0.7931	1.1399	0.5636	0.047*	
H33C	0.839	1.0118	0.5782	0.047*	
C34	1.0899 (4)	0.6077 (4)	0.6418 (3)	0.0251 (10)	
C35	1.1166 (4)	0.5382 (4)	0.7091 (3)	0.0285 (11)	
C36	1.1433 (4)	0.4258 (4)	0.7025 (3)	0.0335 (11)	
H36	1.1591	0.3774	0.7471	0.044*	
C37	1.1465 (4)	0.3850 (4)	0.6306 (3)	0.0327 (12)	
C38	1.1286 (4)	0.4567 (4)	0.5636 (3)	0.0308 (11)	
H38	1.1363	0.429	0.5145	0.04*	
C39	1.0993 (4)	0.5699 (4)	0.5670 (3)	0.0263 (10)	
C40	1.1198 (6)	0.5791 (5)	0.7860 (4)	0.0409 (13)	
H40A	1.1826	0.6167	0.7722	0.053*	
H40B	1.144	0.518	0.8239	0.053*	
H40C	1.035	0.6288	0.8111	0.053*	
C41	1.1681 (6)	0.2642 (4)	0.6278 (4)	0.0447 (14)	
H41A	1.0854	0.2524	0.6445	0.058*	
H41B	1.2198	0.2224	0.6645	0.058*	
H41C	1.2129	0.2409	0.5727	0.058*	
C42	1.0835 (5)	0.6465 (5)	0.4932 (3)	0.0356 (12)	
H42A	0.9999	0.7033	0.5065	0.046*	
H42B	1.0891	0.6065	0.4487	0.046*	
H42C	1.1513	0.679	0.4771	0.046*	
O3	0.0873 (4)	0.7653 (5)	1.0168 (3)	0.0734 (15)	
C43	−0.0328 (7)	0.8164 (9)	1.0722 (5)	0.086 (3)	
D43A	−0.0682	0.8929	1.0547	0.112*	
D43B	−0.0216	0.8134	1.1268	0.112*	
C44	−0.1204 (9)	0.7577 (10)	1.0733 (7)	0.100 (3)	
D44A	−0.1681	0.7896	1.0321	0.129*	
D44B	−0.1823	0.7596	1.127	0.129*	
C45	−0.0341 (12)	0.6449 (9)	1.0546 (8)	0.111 (4)	
D45A	−0.0705	0.6081	1.0275	0.145*	
D45B	−0.0153	0.6018	1.1037	0.145*	
C46	0.0864 (10)	0.6674 (9)	0.9966 (6)	0.098 (3)	
D46A	0.1649	0.6084	1.0027	0.127*	
D46B	0.0832	0.6722	0.9399	0.127*	

### Atomic displacement parameters (Å^2^)

**Table:**

	*U*^11^	*U*^22^	*U*^33^	*U*^12^	*U*^13^	*U*^23^
W1	0.01818 (9)	0.02470 (11)	0.02314 (10)	−0.00853 (6)	−0.00253 (6)	−0.00466 (7)
Cl1	0.0302 (6)	0.0598 (9)	0.0275 (6)	−0.0210 (6)	−0.0064 (5)	−0.0024 (6)
Cl2	0.0273 (6)	0.0348 (7)	0.0563 (8)	−0.0127 (5)	0.0022 (5)	−0.0182 (7)
N1	0.0211 (19)	0.031 (3)	0.048 (3)	−0.0105 (17)	−0.0039 (18)	0.006 (2)
O1	0.039 (2)	0.036 (2)	0.103 (4)	−0.0160 (18)	−0.001 (2)	−0.018 (3)
N2	0.030 (2)	0.067 (3)	0.040 (3)	−0.028 (2)	−0.009 (2)	0.004 (3)
O2	0.063 (3)	0.149 (5)	0.038 (3)	−0.065 (3)	−0.026 (2)	0.028 (3)
C1	0.025 (2)	0.024 (2)	0.023 (2)	−0.0101 (18)	−0.0046 (18)	−0.002 (2)
N3	0.0227 (18)	0.039 (2)	0.028 (2)	−0.0139 (17)	−0.0039 (16)	−0.006 (2)
C2	0.028 (3)	0.078 (4)	0.034 (3)	−0.031 (3)	0.000 (2)	−0.005 (3)
C3	0.027 (2)	0.076 (4)	0.035 (3)	−0.029 (3)	−0.003 (2)	−0.008 (3)
N4	0.0233 (18)	0.039 (2)	0.027 (2)	−0.0157 (17)	−0.0064 (16)	−0.001 (2)
C4	0.022 (2)	0.035 (3)	0.020 (2)	−0.0131 (19)	−0.0006 (17)	−0.003 (2)
C5	0.030 (2)	0.036 (3)	0.024 (3)	−0.015 (2)	0.0027 (19)	−0.007 (2)
C6	0.032 (3)	0.048 (3)	0.028 (3)	−0.010 (2)	−0.001 (2)	−0.004 (3)
C7	0.029 (2)	0.065 (4)	0.027 (3)	−0.017 (3)	−0.002 (2)	−0.012 (3)
C8	0.033 (3)	0.049 (3)	0.038 (3)	−0.019 (2)	0.004 (2)	−0.022 (3)
C9	0.025 (2)	0.036 (3)	0.032 (3)	−0.014 (2)	0.0021 (19)	−0.009 (2)
C10	0.053 (3)	0.030 (3)	0.037 (3)	−0.011 (2)	0.000 (2)	−0.007 (3)
C11	0.045 (3)	0.108 (6)	0.035 (3)	−0.022 (4)	−0.009 (3)	−0.021 (4)
C12	0.038 (3)	0.037 (3)	0.050 (4)	−0.007 (2)	0.003 (3)	−0.006 (3)
C13	0.018 (2)	0.037 (3)	0.029 (3)	−0.0124 (19)	−0.0073 (18)	−0.004 (2)
C14	0.023 (2)	0.036 (3)	0.040 (3)	−0.010 (2)	−0.012 (2)	−0.001 (3)
C15	0.026 (2)	0.050 (4)	0.041 (3)	−0.006 (2)	−0.008 (2)	−0.019 (3)
C16	0.025 (2)	0.057 (4)	0.031 (3)	−0.013 (2)	−0.006 (2)	−0.011 (3)
C17	0.027 (2)	0.046 (3)	0.034 (3)	−0.015 (2)	−0.008 (2)	0.003 (3)
C18	0.017 (2)	0.034 (3)	0.034 (3)	−0.0109 (18)	−0.0037 (18)	−0.007 (2)
C19	0.051 (3)	0.038 (3)	0.060 (4)	−0.011 (3)	−0.023 (3)	−0.004 (3)
C20	0.037 (3)	0.092 (5)	0.036 (3)	−0.016 (3)	−0.011 (2)	−0.014 (4)
C21	0.034 (3)	0.036 (3)	0.050 (3)	−0.012 (2)	−0.012 (2)	−0.005 (3)
C22	0.025 (2)	0.023 (2)	0.019 (2)	−0.0075 (18)	−0.0062 (17)	−0.005 (2)
N5	0.0192 (17)	0.023 (2)	0.025 (2)	−0.0072 (14)	−0.0049 (15)	−0.0036 (18)
C23	0.022 (2)	0.029 (3)	0.028 (2)	−0.0136 (18)	−0.0053 (18)	−0.001 (2)
C24	0.020 (2)	0.033 (3)	0.029 (3)	−0.0113 (19)	−0.0028 (18)	−0.007 (2)
N6	0.0208 (17)	0.021 (2)	0.024 (2)	−0.0065 (15)	−0.0043 (15)	−0.0034 (18)
C25	0.020 (2)	0.022 (2)	0.030 (3)	−0.0092 (17)	−0.0040 (18)	−0.006 (2)
C26	0.027 (2)	0.031 (3)	0.027 (3)	−0.0130 (19)	−0.0074 (19)	−0.003 (2)
C27	0.038 (3)	0.034 (3)	0.027 (3)	−0.015 (2)	−0.006 (2)	−0.008 (2)
C28	0.032 (2)	0.028 (3)	0.035 (3)	−0.012 (2)	−0.001 (2)	−0.007 (2)
C29	0.030 (2)	0.025 (3)	0.031 (3)	−0.0079 (19)	−0.004 (2)	0.000 (2)
C30	0.025 (2)	0.026 (3)	0.026 (2)	−0.0119 (19)	−0.0013 (18)	−0.003 (2)
C31	0.054 (3)	0.041 (3)	0.033 (3)	−0.009 (3)	−0.018 (3)	−0.005 (3)
C32	0.054 (3)	0.037 (3)	0.052 (4)	−0.013 (3)	−0.004 (3)	−0.017 (3)
C33	0.048 (3)	0.029 (3)	0.029 (3)	−0.011 (2)	−0.011 (2)	0.000 (2)
C34	0.0168 (19)	0.023 (2)	0.031 (3)	−0.0033 (17)	−0.0021 (18)	−0.004 (2)
C35	0.024 (2)	0.028 (3)	0.031 (3)	−0.0050 (19)	−0.0072 (19)	−0.001 (2)
C36	0.029 (2)	0.028 (3)	0.040 (3)	−0.004 (2)	−0.011 (2)	0.000 (3)
C37	0.029 (2)	0.023 (3)	0.044 (3)	−0.0068 (19)	−0.007 (2)	−0.007 (3)
C38	0.029 (2)	0.030 (3)	0.031 (3)	−0.009 (2)	−0.002 (2)	−0.010 (2)
C39	0.020 (2)	0.027 (3)	0.029 (3)	−0.0057 (18)	−0.0021 (18)	−0.007 (2)
C40	0.057 (3)	0.030 (3)	0.040 (3)	−0.011 (2)	−0.023 (3)	0.000 (3)
C41	0.051 (3)	0.026 (3)	0.055 (4)	−0.008 (2)	−0.017 (3)	−0.006 (3)
C42	0.042 (3)	0.036 (3)	0.028 (3)	−0.011 (2)	−0.007 (2)	−0.006 (3)
O3	0.052 (3)	0.111 (5)	0.061 (3)	−0.040 (3)	−0.003 (2)	−0.008 (3)
C43	0.061 (4)	0.134 (8)	0.074 (5)	−0.039 (5)	−0.015 (4)	−0.023 (6)
C44	0.083 (6)	0.138 (10)	0.093 (7)	−0.063 (6)	−0.026 (5)	0.020 (7)
C45	0.138 (9)	0.084 (7)	0.124 (9)	−0.066 (7)	−0.037 (8)	0.037 (7)
C46	0.107 (7)	0.088 (7)	0.084 (7)	−0.017 (6)	−0.023 (6)	−0.008 (6)

### Geometric parameters (Å, °)

**Table:**

W1—N2	1.815 (5)	N5—C25	1.449 (5)
W1—N1	1.856 (4)	C23—C24	1.332 (7)
W1—C1	2.278 (4)	C23—H23	0.93
W1—C22	2.284 (4)	C24—N6	1.392 (6)
W1—Cl2	2.4315 (12)	C24—H24	0.93
W1—Cl1	2.4449 (13)	N6—C34	1.457 (6)
N1—O1	1.143 (6)	C25—C30	1.391 (7)
N2—O2	1.188 (6)	C25—C26	1.401 (7)
C1—N3	1.364 (6)	C26—C27	1.392 (7)
C1—N4	1.369 (6)	C26—C31	1.490 (8)
N3—C2	1.390 (6)	C27—C28	1.385 (8)
N3—C4	1.453 (6)	C27—H27	0.93
C2—C3	1.319 (8)	C28—C29	1.395 (7)
C2—H2	0.93	C28—C32	1.509 (7)
C3—N4	1.387 (6)	C29—C30	1.393 (7)
C3—H3	0.93	C29—H29	0.93
N4—C13	1.459 (6)	C30—C33	1.497 (7)
C4—C5	1.389 (7)	C31—H31A	0.96
C4—C9	1.392 (7)	C31—H31B	0.96
C5—C6	1.393 (7)	C31—H31C	0.96
C5—C10	1.505 (7)	C32—H32A	0.96
C6—C7	1.382 (8)	C32—H32B	0.96
C6—H6	0.93	C32—H32C	0.96
C7—C8	1.385 (9)	C33—H33A	0.96
C7—C11	1.508 (8)	C33—H33B	0.96
C8—C9	1.379 (8)	C33—H33C	0.96
C8—H8	0.93	C34—C35	1.388 (7)
C9—C12	1.507 (8)	C34—C39	1.398 (7)
C10—H10A	0.96	C35—C36	1.402 (7)
C10—H10B	0.96	C35—C40	1.504 (7)
C10—H10C	0.96	C36—C37	1.393 (7)
C11—H11A	0.96	C36—H36	0.93
C11—H11B	0.96	C37—C38	1.379 (8)
C11—H11C	0.96	C37—C41	1.513 (7)
C12—H12A	0.96	C38—C39	1.399 (7)
C12—H12B	0.96	C38—H38	0.93
C12—H12C	0.96	C39—C42	1.496 (7)
C13—C18	1.377 (7)	C40—H40A	0.96
C13—C14	1.403 (7)	C40—H40B	0.96
C14—C15	1.396 (8)	C40—H40C	0.96
C14—C19	1.500 (8)	C41—H41A	0.96
C15—C16	1.384 (8)	C41—H41B	0.96
C15—H15	0.93	C41—H41C	0.96
C16—C17	1.386 (8)	C42—H42A	0.96
C16—C20	1.529 (8)	C42—H42B	0.96
C17—C18	1.392 (7)	C42—H42C	0.96
C17—H17	0.93	O3—C46	1.376 (11)
C18—C21	1.513 (7)	O3—C43	1.403 (9)
C19—H19A	0.96	C43—C44	1.454 (12)
C19—H19B	0.96	C43—D43A	0.97
C19—H19C	0.96	C43—D43B	0.97
C20—H20A	0.96	C44—C45	1.467 (15)
C20—H20B	0.96	C44—D44A	0.97
C20—H20C	0.96	C44—D44B	0.97
C21—H21A	0.96	C45—C46	1.526 (14)
C21—H21B	0.96	C45—D45A	0.97
C21—H21C	0.96	C45—D45B	0.97
C22—N5	1.370 (6)	C46—D46A	0.97
C22—N6	1.370 (5)	C46—D46B	0.97
N5—C23	1.390 (5)		
			
N2—W1—N1	89.2 (2)	C22—N5—C25	130.6 (3)
N2—W1—C1	95.23 (17)	C23—N5—C25	117.6 (3)
N1—W1—C1	89.24 (16)	C24—C23—N5	107.3 (4)
N2—W1—C22	89.32 (17)	C24—C23—H23	126.3
N1—W1—C22	95.56 (16)	N5—C23—H23	126.3
C1—W1—C22	173.44 (18)	C23—C24—N6	106.8 (4)
N2—W1—Cl2	92.77 (17)	C23—C24—H24	126.6
N1—W1—Cl2	171.34 (11)	N6—C24—H24	126.6
C1—W1—Cl2	82.19 (12)	C22—N6—C24	111.4 (4)
C22—W1—Cl2	92.89 (11)	C22—N6—C34	128.5 (4)
N2—W1—Cl1	171.32 (13)	C24—N6—C34	119.8 (3)
N1—W1—Cl1	91.09 (15)	C30—C25—C26	122.7 (4)
C1—W1—Cl1	93.45 (12)	C30—C25—N5	118.5 (4)
C22—W1—Cl1	82.02 (12)	C26—C25—N5	118.4 (4)
Cl2—W1—Cl1	88.23 (5)	C27—C26—C25	117.1 (5)
O1—N1—W1	174.1 (4)	C27—C26—C31	120.6 (4)
O2—N2—W1	173.0 (4)	C25—C26—C31	122.3 (4)
N3—C1—N4	102.8 (3)	C28—C27—C26	122.4 (5)
N3—C1—W1	126.0 (3)	C28—C27—H27	118.8
N4—C1—W1	131.2 (3)	C26—C27—H27	118.8
C1—N3—C2	111.8 (4)	C27—C28—C29	118.3 (4)
C1—N3—C4	129.8 (4)	C27—C28—C32	121.0 (5)
C2—N3—C4	118.2 (4)	C29—C28—C32	120.7 (5)
C3—C2—N3	106.6 (4)	C30—C29—C28	121.9 (5)
C3—C2—H2	126.7	C30—C29—H29	119.1
N3—C2—H2	126.7	C28—C29—H29	119.1
C2—C3—N4	107.6 (4)	C25—C30—C29	117.5 (4)
C2—C3—H3	126.2	C25—C30—C33	121.7 (4)
N4—C3—H3	126.2	C29—C30—C33	120.8 (5)
C1—N4—C3	111.2 (4)	C26—C31—H31A	109.5
C1—N4—C13	131.2 (4)	C26—C31—H31B	109.5
C3—N4—C13	117.7 (4)	H31A—C31—H31B	109.5
C5—C4—C9	121.9 (4)	C26—C31—H31C	109.5
C5—C4—N3	118.6 (4)	H31A—C31—H31C	109.5
C9—C4—N3	118.9 (4)	H31B—C31—H31C	109.5
C4—C5—C6	117.7 (4)	C28—C32—H32A	109.5
C4—C5—C10	122.0 (5)	C28—C32—H32B	109.5
C6—C5—C10	120.3 (5)	H32A—C32—H32B	109.5
C7—C6—C5	122.0 (5)	C28—C32—H32C	109.5
C7—C6—H6	119	H32A—C32—H32C	109.5
C5—C6—H6	119	H32B—C32—H32C	109.5
C6—C7—C8	118.1 (5)	C30—C33—H33A	109.5
C6—C7—C11	121.1 (6)	C30—C33—H33B	109.5
C8—C7—C11	120.8 (5)	H33A—C33—H33B	109.5
C9—C8—C7	122.3 (5)	C30—C33—H33C	109.5
C9—C8—H8	118.8	H33A—C33—H33C	109.5
C7—C8—H8	118.8	H33B—C33—H33C	109.5
C8—C9—C4	117.9 (5)	C35—C34—C39	123.1 (4)
C8—C9—C12	120.1 (5)	C35—C34—N6	118.0 (4)
C4—C9—C12	121.9 (5)	C39—C34—N6	118.7 (5)
C5—C10—H10A	109.5	C34—C35—C36	117.2 (5)
C5—C10—H10B	109.5	C34—C35—C40	122.5 (5)
H10A—C10—H10B	109.5	C36—C35—C40	120.3 (5)
C5—C10—H10C	109.5	C37—C36—C35	121.6 (5)
H10A—C10—H10C	109.5	C37—C36—H36	119.2
H10B—C10—H10C	109.5	C35—C36—H36	119.2
C7—C11—H11A	109.5	C38—C37—C36	118.8 (5)
C7—C11—H11B	109.5	C38—C37—C41	121.5 (5)
H11A—C11—H11B	109.5	C36—C37—C41	119.8 (5)
C7—C11—H11C	109.5	C37—C38—C39	122.2 (5)
H11A—C11—H11C	109.5	C37—C38—H38	118.9
H11B—C11—H11C	109.5	C39—C38—H38	118.9
C9—C12—H12A	109.5	C34—C39—C38	116.9 (5)
C9—C12—H12B	109.5	C34—C39—C42	121.4 (4)
H12A—C12—H12B	109.5	C38—C39—C42	121.7 (4)
C9—C12—H12C	109.5	C35—C40—H40A	109.5
H12A—C12—H12C	109.5	C35—C40—H40B	109.5
H12B—C12—H12C	109.5	H40A—C40—H40B	109.5
C18—C13—C14	123.1 (4)	C35—C40—H40C	109.5
C18—C13—N4	118.6 (4)	H40A—C40—H40C	109.5
C14—C13—N4	117.7 (5)	H40B—C40—H40C	109.5
C15—C14—C13	116.5 (5)	C37—C41—H41A	109.5
C15—C14—C19	120.4 (5)	C37—C41—H41B	109.5
C13—C14—C19	123.0 (5)	H41A—C41—H41B	109.5
C16—C15—C14	122.1 (5)	C37—C41—H41C	109.5
C16—C15—H15	119	H41A—C41—H41C	109.5
C14—C15—H15	119	H41B—C41—H41C	109.5
C15—C16—C17	119.0 (5)	C39—C42—H42A	109.5
C15—C16—C20	120.0 (5)	C39—C42—H42B	109.5
C17—C16—C20	121.0 (6)	H42A—C42—H42B	109.5
C16—C17—C18	121.3 (5)	C39—C42—H42C	109.5
C16—C17—H17	119.4	H42A—C42—H42C	109.5
C18—C17—H17	119.4	H42B—C42—H42C	109.5
C13—C18—C17	118.0 (4)	C46—O3—C43	108.7 (6)
C13—C18—C21	121.3 (5)	O3—C43—C44	107.3 (8)
C17—C18—C21	120.7 (5)	O3—C43—D43A	110.3
C14—C19—H19A	109.5	C44—C43—D43A	110.3
C14—C19—H19B	109.5	O3—C43—D43B	110.3
H19A—C19—H19B	109.5	C44—C43—D43B	110.3
C14—C19—H19C	109.5	D43A—C43—D43B	108.5
H19A—C19—H19C	109.5	C43—C44—C45	104.5 (8)
H19B—C19—H19C	109.5	C43—C44—D44A	110.9
C16—C20—H20A	109.5	C45—C44—D44A	110.9
C16—C20—H20B	109.5	C43—C44—D44B	110.9
H20A—C20—H20B	109.5	C45—C44—D44B	110.9
C16—C20—H20C	109.5	D44A—C44—D44B	108.9
H20A—C20—H20C	109.5	C44—C45—C46	100.1 (8)
H20B—C20—H20C	109.5	C44—C45—D45A	111.8
C18—C21—H21A	109.5	C46—C45—D45A	111.8
C18—C21—H21B	109.5	C44—C45—D45B	111.8
H21A—C21—H21B	109.5	C46—C45—D45B	111.8
C18—C21—H21C	109.5	D45A—C45—D45B	109.5
H21A—C21—H21C	109.5	O3—C46—C45	107.8 (8)
H21B—C21—H21C	109.5	O3—C46—D46A	110.1
N5—C22—N6	103.2 (3)	C45—C46—D46A	110.1
N5—C22—W1	130.8 (3)	O3—C46—D46B	110.1
N6—C22—W1	125.8 (3)	C45—C46—D46B	110.1
C22—N5—C23	111.2 (3)	D46A—C46—D46B	108.5

### Hydrogen-bond geometry (Å, °)

**Table:**

*D*—H···*A*	*D*—H	H···*A*	*D*···*A*	*D*—H···*A*
C2—H2···O3	0.93	2.40	3.320 (7)	172
